# N6-methyladenosine (m6A) RNA methylation mediated by methyltransferase complex subunit WTAP regulates amelogenesis

**DOI:** 10.1016/j.jbc.2022.102715

**Published:** 2022-11-17

**Authors:** Furong Xie, Xueqin Zhu, Xiao Liu, Hui Chen, Jun Wang

**Affiliations:** Department of Pediatric Dentistry, Shanghai Ninth People’s Hospital, Shanghai Jiao Tong University School of Medicine, College of Stomatology, Shanghai Jiao Tong University, National Center for Stomatology, National Clinical Research Center for Oral Diseases, Shanghai Key Laboratory of Stomatology, Shanghai Research Institute of Stomatology, Shanghai, China

**Keywords:** tooth development, enamel, WTAP, m6A methylation, epigenetic regulation, ALC, ameloblast-lineage cell, cKO, conditional KO, IEE, inner enamel epithelium

## Abstract

N6-methyladenosine (m6A) RNA methylation, one of the most widespread posttranscriptional modifications in eukaryotes, plays crucial roles in various developmental processes. The m6A modification process is catalyzed by a methyltransferase complex that includes Wilms tumor 1-associated protein (WTAP) as a key component. Whether the development of dental enamel is regulated by m6A RNA methylation in mammals remains unclear. Here, we reveal that WTAP is widely expressed from the early stage of tooth development. Specific inactivation of *Wtap* in mouse enamel epithelium by the Cre/loxp system leads to serious developmental defects in amelogenesis. In *Wtap* conditional KO mice, we determined that the differentiation of enamel epithelial cells into mature ameloblasts at the early stages of enamel development is affected. Mechanistically, loss of *Wtap* inhibits the expression of Sonic hedgehog (SHH), which plays an important role in the generation of ameloblasts from stem cells. Together, our findings provide new insights into the functional role of WTAP-mediated m6A methylation in amelogenesis in mammals.

As the most mineralized tissue in mammal, dental enamel provides maximum durability that allows teeth to function as weapons and/or tools as well as for food processing. The development and mineralization of enamel is a complex and elaborate process, which is tightly regulated by ameloblasts. Tooth development sequentially undergoes bud stage, cap stage, early and late bell stage. At bell stage, ameloblast differentiate from inner enamel epithelium (IEE) (1) and possess a life cycle of several stages including presecretory ameloblasts, secretory ameloblasts, and mature ameloblasts. Secretory ameloblasts are columnar, tall, polarized cells, which can secret extracellular matrix proteins such as amelogenin (AMELX) (2, 3). The fine mechanisms under the alteration from dental epithelial stem cells to differentiated ameloblasts that lead to enamel formation remains unknown.

N6-methyladenosine (m6A), which was first discovered in 1970s (4), is the most abundant internal mRNA modification. It is a key determinant of posttranscriptional mRNA regulation and function in the regulation in cell fate determination. In mammals, m6A methylation is mediated by the methyltransferase complex, which is composed of METTL3 (methyltransferase-like 3), METTL14, and subunit wilms tumor 1-associated protein (WTAP), KIAA1429, RNA-binding motif protein 15 (RBM15), and its paralog (RBM15B) (5). Nevertheless, methylation is erased by the demethylases fat mass and obesity-associated (FTO) and alkB homolog5 (ALKBH5) (6). At molecular level, m6A modification influences the mRNA metabolisms, including regulating RNA stability (7) and RNA splicing (8), as well as mRNA translation efficiency (9, 10), thus affect the cellular function, identity, and stemness of their residing cells. Many important biological processes are known to be regulated by m6A, including cell fate determination (11) and embryonic development (12, 13). Mammalian WTAP was first identified as protein associated with Wilms’tumor-1 (WT1) (14). Widely, KO of *Wtap* in mice will lead to embryonic death between embryonic day 6.5 to 10.5 and show serious defects in cell proliferation, which in turn leads to defects in endoderm and mesoderm formation (15, 16). WTAP is also a regulatory subunit of the RNA N6-methyladenosine methyltransferase, plays an important enzymatic role in METTL3-METTL14-WTAP methyltransferases complex (17). Recently, many studies proved that WTAP regulated dynamic m6A level, which can not only determine the fate of stem cells and regulate mammalian development but also be involved in carcinogenesis (18, 19).

In this study, *Wtap* conditional KO (cKO) mutant mice were generated. Loss of m6A by inactivation of *Wtap* led to severe amelogenesis imperfecta, which reveals the critical functions of m6A modification and its binding protein WTAP in dental enamel development.

## Results

### WTAP is widely expressed at early stage of tooth development

**Supplemental Figure S1:**
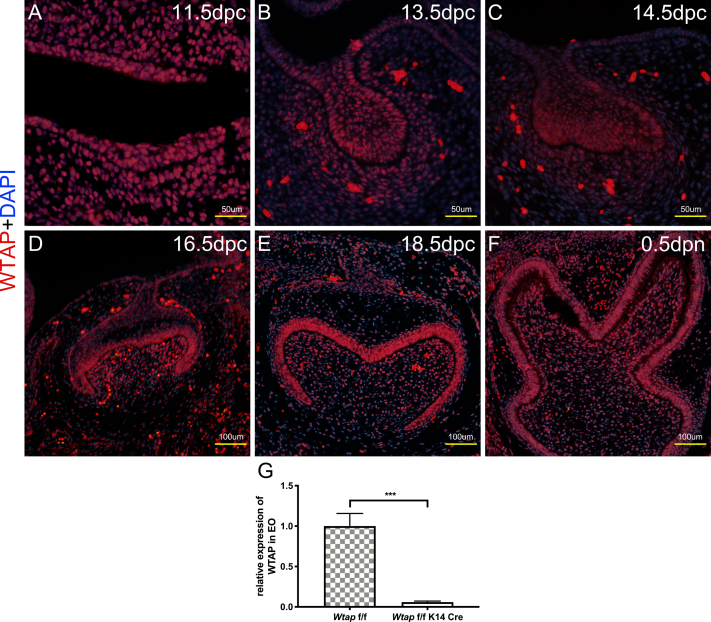


**Supplemental Figure S2:**
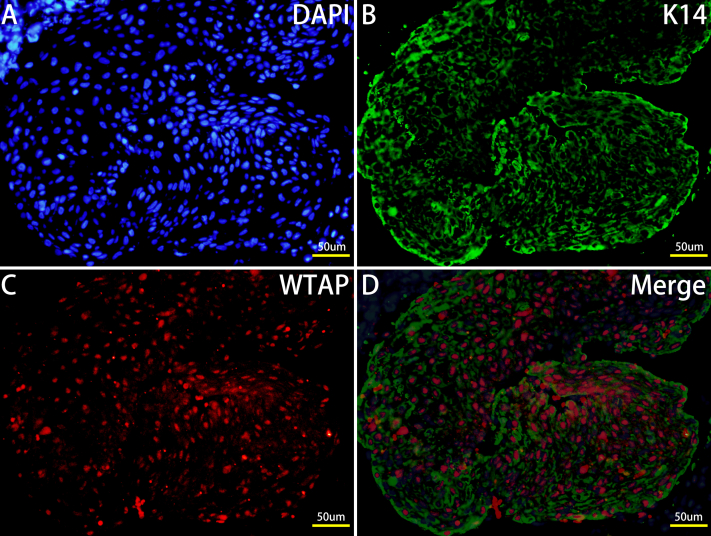


In order to study the role of WTAP in tooth development, we firstly analyzed the expression pattern of WTAP in mice and human sample. At E11.5 (dental lamina), E13.5 (bud stage), E14.5 (cap stage), E16.5 (early bell stage), E18.5 (late bell stage), and P0 (late bell stage) mice, we detected ubiquitous occupation of WTAP in IEE by immunostaining (Fig. S1, *A–F*), indicating that WTAP might be involved in enamel development. In human reduced dental epithelium of unerupted third molar tooth, we also detected strong expression of WTAP protein (Fig. S2), proving that expression pattern of WTAP was similar between mice and human.

### *Wtap* cKO mice displayed amelogenesis imperfecta phenotypes

**Figure 1:**
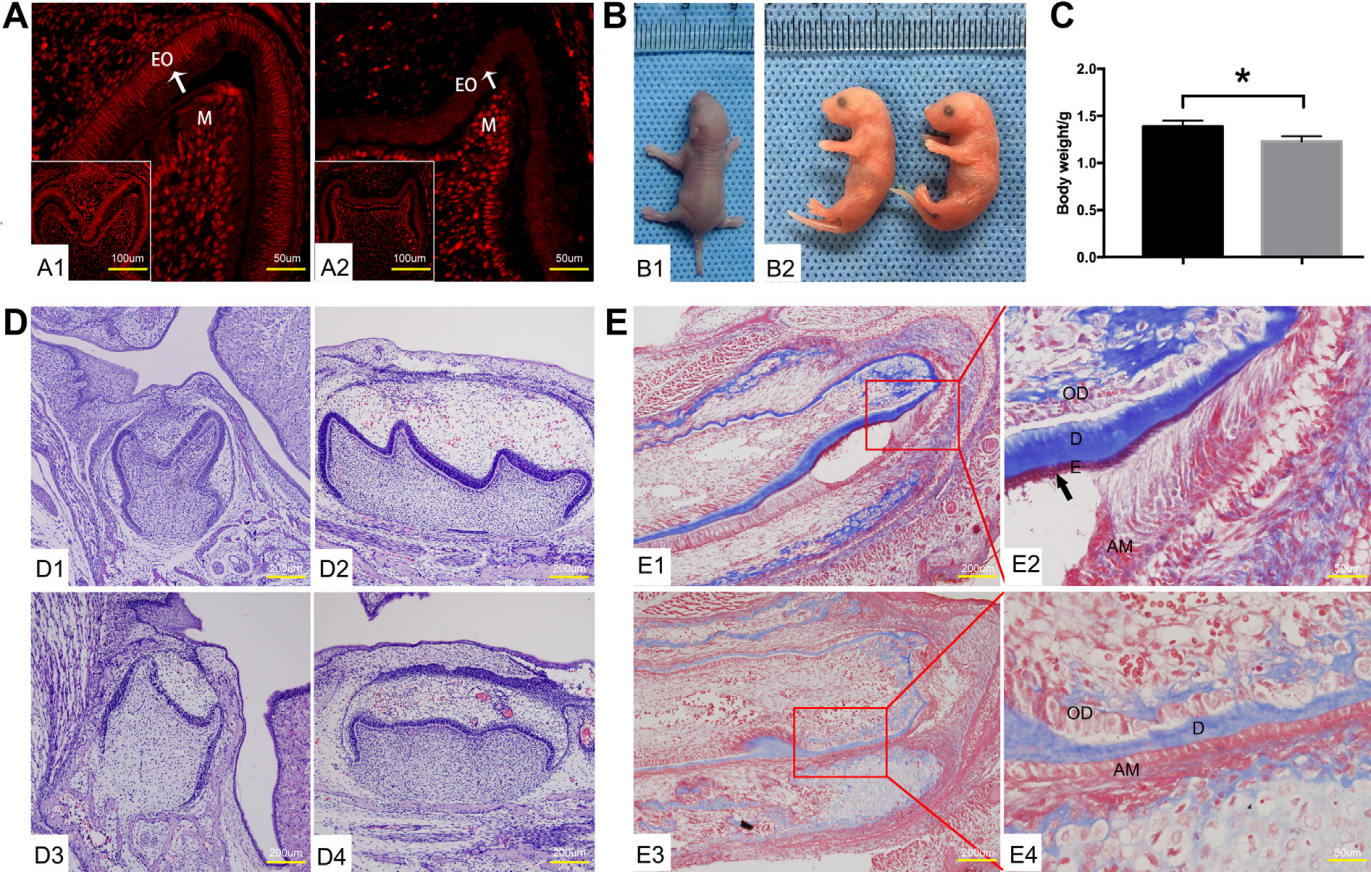
**KO of *Wtap* in dental epithelial cells resulted in enamel malformation.***A*, expression of WTAP protein in dental germ of newborn (0.5dpn) mice were detected *via* immunofluorescence staining (*A1*: control mice; *A2*: cKO mice; EO: enamel organ; M: dental mesenchymal tissue; *white arrow*: inner enamel epithelial cells). *B*, photo of control and cKO mice on birth day (*B1*: control mice; *B2*: cKO mice). *C*, the weight of cKO mice was significantly lower compared with control mice. (∗*p* < 0.05). *D*, observation of mandibular mandibular first molar *via* H&E staining. (*D1*; *D2*: mandibular first molar of control mice; *D3*; *D4*: mandibular first molar from cKO mice). *E*, enamel formation of mandibular incisors was observed *via* masson staining (*E1*: picture of tip of mandibular incisor from control mice; *E2*: high magnification of *red* frame part from *E1*; *E3*: picture of tip of mandibular incisor from cKO mice; *E4*: high magnification of *red* frame part from *E2*. *Black arrow*: deposition of enamel matrix).

**Supplemental Figure S3:**
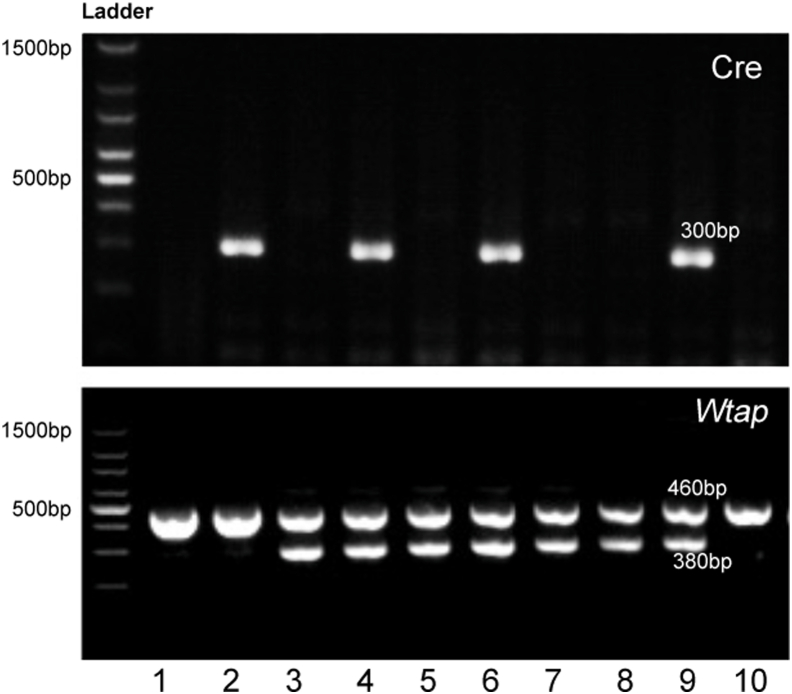


In order to study the biological function of the m6A writer WTAP in enamel development, *Wtap* floxed mice bearing loxP sites flanking exons 4 to 5 of the *Wtap* gene were obtained from professor Minghan Tong’s Laboratory. The precise methods were previously described by Tong’s group (20). The resulting *Wtap*^flox/flox^ mice were crossed with K14-cre transgenic mice to generate *Wtap* cKO mice (genotype *Wtap*^flox/flox^; K14-Cre, named as cKO) (Fig. S3). Littermates of the cKO mice identified with *Wtap*^flox/flox^ genotypes were used as control subjects. Immunohistochemical staining (Fig. 1*A*, and S1*G*) results showed that the cKO mice failed to produce WTAP protein in enamel organ tissue. Nevertheless, WTAP protein remained unperturbed in other dental germ tissues, such as dental mesenchyme.

**Figure 2:**
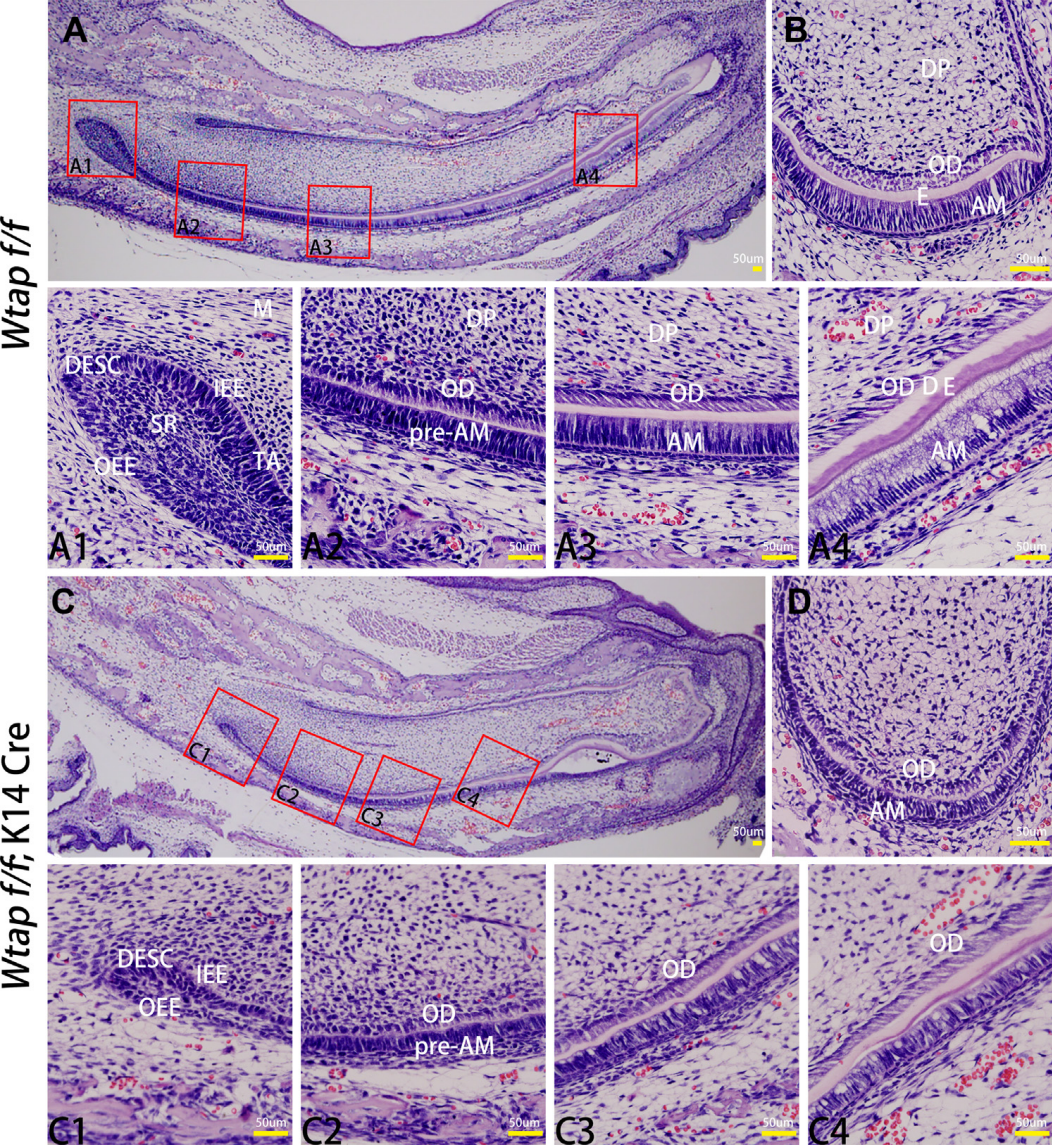
***Wtap* deletion in enamel epithelium affected the polarization of ameloblasts.***A*, observation of sagittal histology of mandibular incisor from control mice; *A1–A4*: the high magnification observation results of the *red boxs* in (*A*), respectively(×400). *B*, observation of coronal histology of mandibular incisor from control mice (×400). Transection is made between *A3* and *A4*. *C*, observation of sagittal histology of mandibular incisor from mutant mice; *C1–C4*: the high magnification observation results of the *red boxs* in (*C*), respectively (×400). *D*, observation of coronal histology of mandibular incisor from mutant mice (×400). Transection is made between *C3* and *C4*.

Compared to the controls, cKO mice had a notably decreased body weight at P0 (Fig. 1, *B* and *C*). No obvious phenotypic changes were found in the head, leg, and size by gross observation. However, at P0, we found severely enamel developmental failure in all of cKO mice. The tooth size and enamel thickness were observed largely deceased in both incisors and molars (Figs. 1*D* and 2*C*). The cusps of molars changed to small and blunt (Figs. 1, *D*3 and *D*4). The degree of enamel mineralization in mutant and control teeth was detected by MASSON trichrome staining on P0 incisor. Dentin formation was present on control and cKO mice (Fig. 1*E*). However, enamel was undetectable in the cKO incisor (Figs. 1, *E*3 and *E*4), indicating that disruption of the *Wtap* gene results in enamel malformation during the early developmental stages of amelogenesis.

**Figure 3:**
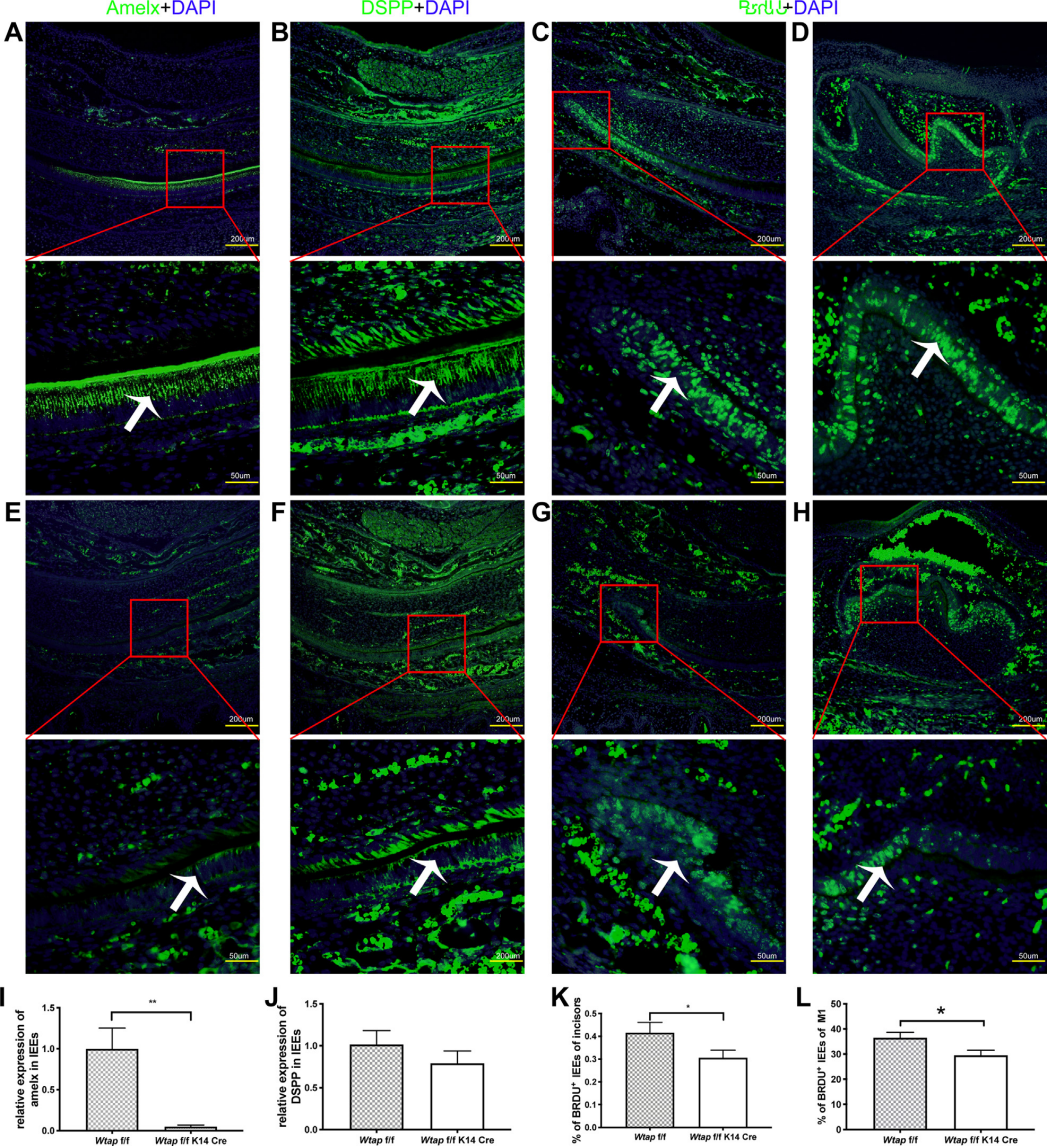
**WTAP promoted differentiation and proliferation of dental inner enamel epithelial cells (IEEs).***A*, expression of AMELX in control mandibular incisor. *B*, expression of DSPP in control mandibular incisor. *C* and *D*, expression of BrdU in control mandibular incisor and molar. *E*, expression of AMELX in mutant mandibular incisor. *F*, expression of DSPP in mutant mandibular incisor. *G* and *H*, expression of BrdU in mutant mandibular incisor and molar. *I* and *J*, statistics analysis result of relative expression of AMELX and DSPP. *K* and *L*, statistics analysis result of relative expression of BrdU in mandibular incisor and first molar respectively. (*White arrow*: IEEs; ∗*p* < 0.05, ∗∗*p* < 0.01).

**Figure 4:**
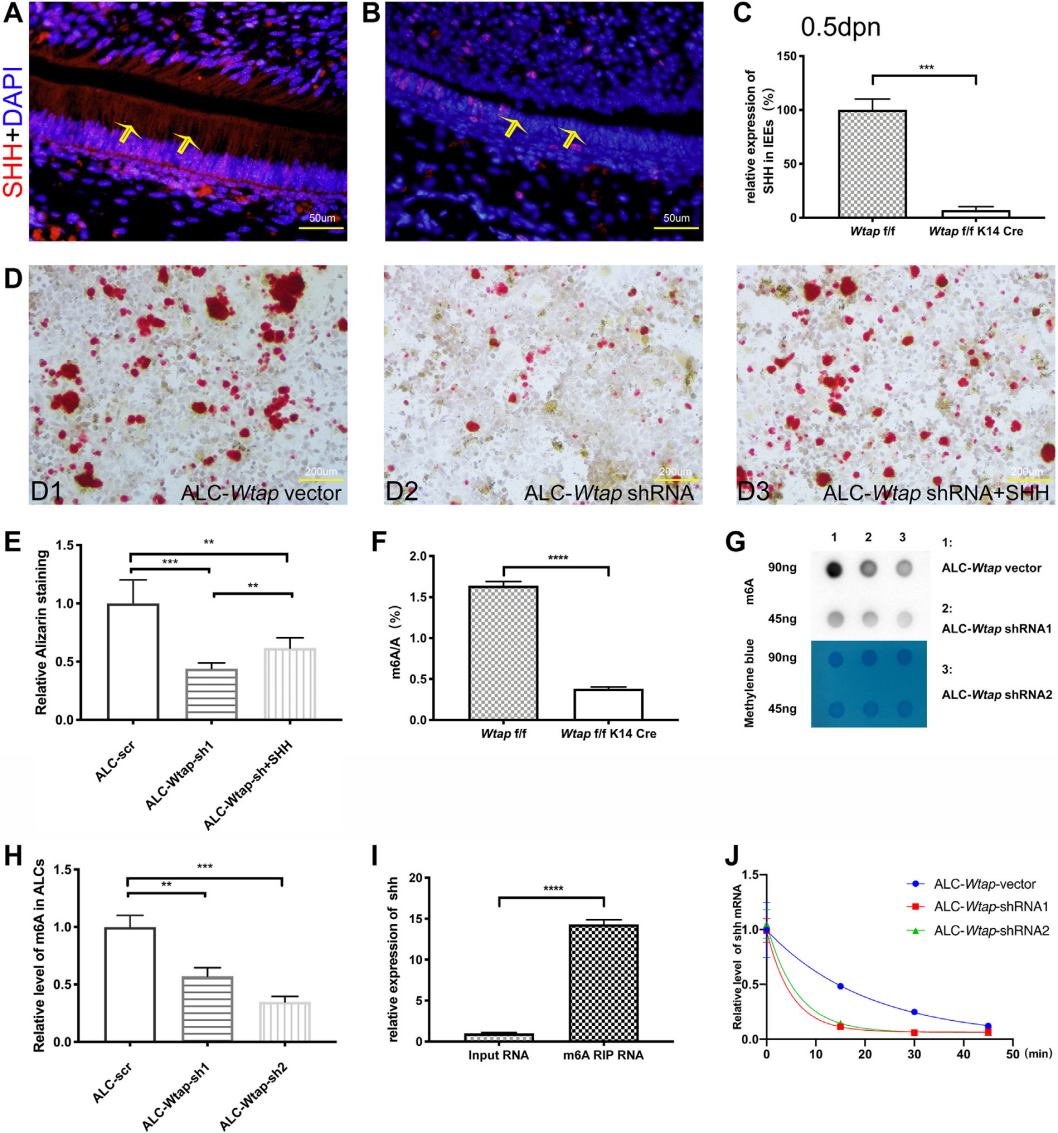
**SHH expression is regulated by WTAP-mediated m6A RNA methylation.***A*, SHH expression in dental IEEs from control mandibular incisor was detected by immunofluorescence staining. *B*, SHH expression in dental IEEs from mutant mandibular incisor was detected by immunofluorescence staining. *C*, results of real-time PCR showed decreased expression of SHH in dental IEEs of mandibular incisor at P0. *D*, recombinant SHH protein rescued differentiation ability of *Wtap* knockdown ALC cells. *E*, quantitative analysis of the differentiation ability of *Wtap* knockdown ALC cells and after recombinant SHH protein treatment. *F*, levels of m6A in dental epithelial tissues of mandibular first molars were detected by UPLC-MS/MS analysis. *G*, dot blot assay showed that levels of m6A in *Wtap* knockdown ALC cells was significantly decreased. *H*, quantitative analysis of m6A level in ALC cells after *Wtap* knockdown. *I*, m6A enrichment in shh mRNA in dental germ at E14.5 by m6A-RIP-qPCR. *J*, quantitative analysis of shh mRNA level after *wtap* knockdown. (∗∗*p* < 0.01; ∗∗∗*p* < 0.001; ∗∗∗∗*p* < 0.0001). ALC, ameloblast-lineage cell; IEE, inner enamel epithelium.

### *Wtap* deletion in enamel epithelium affects the differentiation of ameloblasts at early stages

**Supplemental Figure S4:**
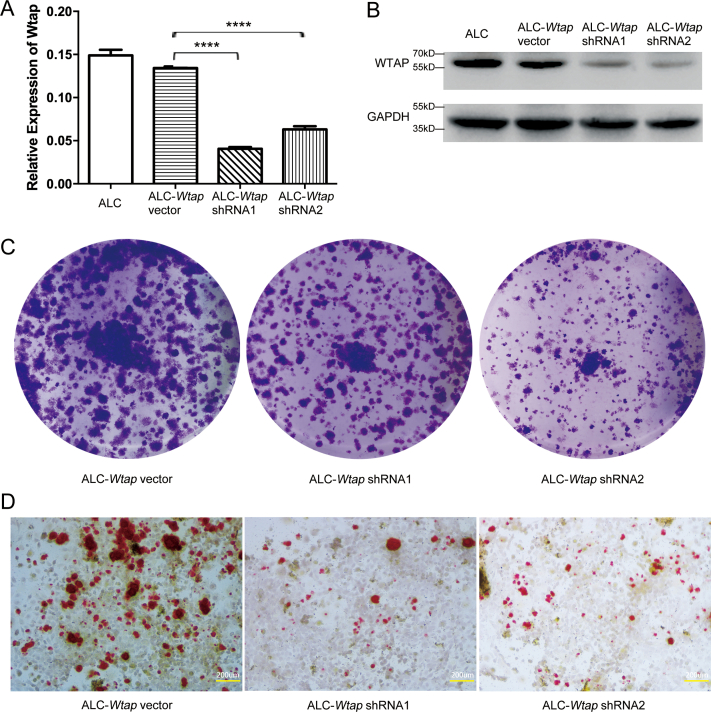


To determine how depletion of *Wtap* affects enamel development, we further assessed the morphologic differences of ameloblasts between cKO and control mice (Fig. 2). Incisors were selected to observe because it can reflect different stages of amelogenesis, including secretory, transition, and maturation phases (21). In control mice, we found morphology of the IEE cells gradually changed from cubic to long columnar in the sagittal section and the cell polarity gradually became obvious with nucleus far away from basement membrane (Fig. 2, *A2–A4*). However, there are no obvious changes in cell morphology of IEE cells in cKO mice (Fig. 2*C*). The IEE cells of cKO mice showed no increase in length and polarization of these cells (Fig. 2, *C2–C4*). The cervical loop was significantly thinner, with fewer cells in cKO mice (Fig. 2*C1*). From the coronal view, more matrix deposited in the junction between IEE cells and odontoblast in WT incisors (Fig. 2*B*), while less in cKO incisors (Fig. 2*D*). *In vitro*, we also found decreased calcium knots formation in *Wtap* knock-down ameloblast-lineage cells (ALCs) (22) which confirmed that WTAP is vital for the proliferation and differentiation of ameloblasts (Fig. S4).

To investigate which stages of ameloblast differentiation has been affected, the genes expression levels of enamel matrix proteins and proteinases, which represent stages of ameloblast cell differentiation, were examined by immunohistochemical staining. We found that AMELX, which is secreted by secretory-stage ameloblasts, was strongly expressed at anterior end of labial side of the incisor in control (Fig. 3*A*). On the contrary, AMELX expression was significantly decreased in the cKO incisor (Fig. 3, *E* and *I*). Interestingly, expression of dentin sialophosphoprotein (DSPP) seemed to have no significant difference between WT and mutant mice (Fig. 3, *B*, *F* and *J*). DSPP was reported to express transiently in presecretory stage ameloblasts, while not expressed in secretory stage. Enamel epithelial cells are postmitotic cells in presecretory stage ameloblasts. The BrdU staining is present in all G2/S cells but not post-mitotic cells. In P0 control incisors, BrdU staining was not found at the anterior end of the labial side but gradually expressed at the posterior region (Fig. 3*C*). In P0 cKO incisor, we observed a decreased pattern of BrdU staining expression at the posterior region (Fig. 3, *G* and *K*). In molar, same expression pattern was found in cKO and control mice (Fig. 3, *D*, *H* and *L*). Taken together, the *in vivo* evidence indicates a striking phenotype of abnormal enamel development, resulting from affected differentiation and proliferation of the enamel epithelium to mature ameloblasts at the early stages of enamel development.

### Loss of *Wtap* deregulated SHH expression and affects ameloblasts differentiation

**Supplemental Figure S6:**
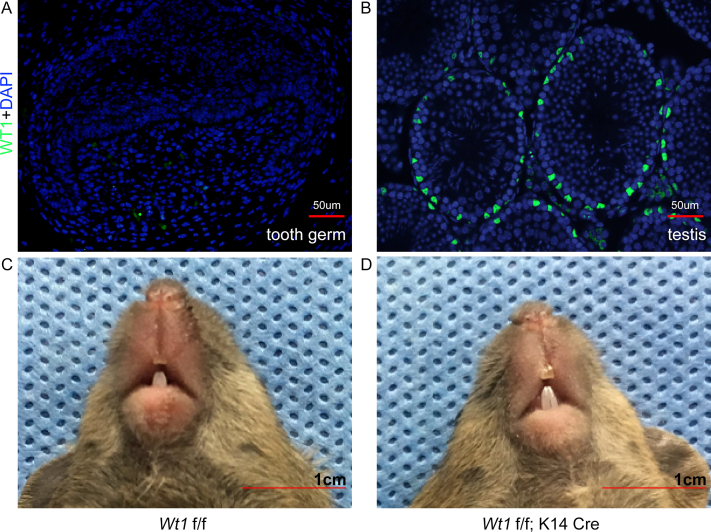


To investigate the potential mechanism, we probed for Sonic hedgehog (SHH), which plays an important role in generation of ameloblasts from stem cells (23). In cKO, expression of SHH in ameloblasts of incisior was decreased (Fig. 4, *A–C*). This decreased expression was confirmed by reverse transcription quantitative PCR (Fig. 4*C*). In 16.5dpc cKO, the tooth embryo develops in the cap stage. We also observed decreased expression of SHH in ameloblasts of molar (Fig. S5, *A–C*). *In vitro*, recombinant SHH protein also can rescue the differentiation ability of *Wtap* knockdown ALC cells (Fig. 4, *D* and *E*). The results indicated that WTAP plays a critical role in regulation of SHH expression in ameloblasts. Previous studies proved that WTAP is an important subunit in the m6A methyltransferase complex and plays a critical role in epitranscriptomic m6A regulation (17). We found that m6A level was notably reduced in cKO dental epithelial tissue of mandibular first molar (Fig. 4*F*). *In vitro*, results of dot blot assay also showed that m6A level is obviously reduced after WTAP deletion in ALC cells (Fig. 4, *G* and *H*). *In vivo*, by performing m6A-RIP-qPCR, we proved m6A enrichment in *Shh* mRNA (Fig. 4*I*). Futhermore, result of mRNA stability assay showed that shh mRNA half-life decreased after *Wtap* KO in ALC cells (Fig. 4*J*), which means shh mRNA stability became weaker. Previous studies also found that WTAP exerts a concentration-dependent inhibitory effect on the expression of WT1-sensitive genes that regulate both mitogenic and survival pathways (24). However, we found that WT1 was hardly expressed in dental epithelial cells and *Wt1* cKO mice displayed normal amelogenesis as well as WT mice (Fig. S6). It is indicated that WTAP affects enamel development not by inhibiting WT1-sensitive genes pathway. Concluded that WTAP mediated ameloblasts differentiation *via* affecting SHH signaling through m6A but not binding to WT1. Then, WTAP may promote enamel development by regulating the expression of SHH protein *via* m6A methylation.

## Discussion

Enamel development involves various stages that are tightly controlled by several key molecules of major signaling pathways expressed in epithelial and mesenchymal cells. Subtle alterations during this complicated process could lead to severe enamel defects in structure, color, and shape. For a long time, it was believed that all the genetic information was stored in the sequence of DNA. However, the differences in tooth morphology between monozygotic twins indicated that other mechanisms exert their effect on gene translation (25, 26). These mechanisms are called epigenetics, which are alterations in gene expression without changes in the DNA sequence. Several studies have proved epigenetics are important in the regulation of tooth development and tooth regeneration (27, 28). Modulations in microRNA, lncRNA, DNA methylation, and chromatin modifications are proved important regulatory mechanisms during tooth development (29, 30, 31, 32, 33, 34, 35). However, RNA modification in regulation of tooth development remains unclear. Here, we used the epithelial cell special marker, keratin 14, to establish the genetic mice lineage to conditionally delete *Wtap* from the initiation of enamel formation. Loss of *Wtap* leads to a severe amelogenesis imperfecta-like phenotype with smaller tooth size and thinner enamel, suggesting the functional importance of WTAP in ameloblasts. WTAP was initially identified as a nuclear protein that specifically interacts with WT1 in the development of mouse embryo (16). However, WT1 is not required for enamel formation in our study. Deletion of *Wt1* in mice did not generate the same phenotype of *Wtap* cKO mice. Hence, we performed dot blot assay, which proved that m6A level is obviously reduced after *Wtap* deletion. The results indicate that WTAP-mediated m6A plays an important role in amelogenesis.

The process of amelogenesis includes four defined stages: presecretory, secretory, transition, and maturation. Hu *et al*.(36) illustrated the changing ameloblast morphologies throughout amelogenesis as viewed histologically. However, in cKO mice, we observed shorter and unpolarized ameloblasts. The cervical loop was significantly thinner. The changing ameloblast morphologies from the root to the cut end cannot be found. The results indicats that WTAP plays a crucial role in maturation of ameloblast. Previous studies have proved that SHH and FGF signaling are required from the initiation stage of tooth development onward. In E11.5 mouse, the molar begin forming, a group of FGF8-positive cells form a rosette-like structure and move toward a SHH-positive cell center (37). Moreover, inhibition of SHH signaling resulted in the abnormality of the growth and invagination of dental epithelium (37). It is also believed that SHH is indispensable for the development of cytoskeleton in ameloblasts, which is critical to maintaining epithelial cell polarity and intercellular communication (38). In cKO mice, expression of SHH in ameloblasts was decreased, indicating that WTAP modulates ameloblast maturation by regulating the expression of SHH.

Previous studies have shown that WTAP-mediated m6A could influence many biological processes. m6A-RIP-qPCR also verified m6A enrichment in shh mRNA in dental tissue. Furthermore, incubation of recombinant SHH partially rescued the capacity of mineralization ability in *Wtap* silenced ameloblasts. Based on these evidences, WTAP was believed to regulate *Shh* expression by maintaining the level of m6A modification. It will be of interest to explore the precise regulating mechanism in the future. Mice lacking WTAP die between embryonic day 6.5 to 10.5 and show dramatic defects in endoderm and mesoderm formation (15, 16). The cKO mice in our study die between postnatal day 1 to 2. It limits our observation of the tooth phenotype in the matured mice at 8 weeks old or later. Inducible gene KO mice should be used in the future study.

Here, we presented some findings demonstrating the importance of m6A in amelogenesis. Firstly, conditional deletion of *Wtap* in ameloblasts led to defective amelogenesis, demonstrating an essential role of WTAP-mediated m6A modification in ameloblast maturation. Secondly, m6A-RIP-qPCR assays revealed that deletion of *Wtap* resulted in downregulation of *Shh*. In conclusion, WTAP plays a critical role in amelogenesis and may regulate the maturation of ameloblasts by modulating expression of SHH and downstream molecules.

## Experimental procedures

### Animal and ethics statement

*Wtap* floxed mice, K14 Cre mice were gifts from Pro. Minghan Tong’s lab (CAS Center for Excellence in Molecular Cell Science, Shanghai Institute of Biochemistry and Cell Biology). All mice were C57BL/6 genetic background and were bred under specific pathogen-free conditions. *Wtap*^f^^lox^^/+^; K14 Cre mice mated with *Wtap*^f^^lox^^/f^^lox^ mice were utilized to obtain *Wtap*^f^^lox^^/f^^lox^; K14 Cre mice embryos. Primers for genotyping were listed in Table S1. This study was approved by the Ethical Review Committee of the Ninth People's Hospital, Shanghai Jiao Tong University School of Medicine (Shanghai, China).

### Histochemical and immunohistochemical analysis

Heads from embryonic or postnatal mice were dissected in PBSand fixed in 4% paraformaldehyde-PBS solution overnight. The tissues were then dehydrated through graded ethanol, embedded in paraffin wax, and sectioned by 4 μm. H&E staining was the first step for observation. Immunofluorescence was performed to identify the expression pattern of key signaling molecule. Firstly, slides were boiled in sodium citrate buffer (10 μM, pH 6.0) for 15 min. When it were cooled in room temperature (RT), PBS with 0.1% Triton X-100 was used to wash the slides. Next, 10% donkey serum and 0.1% Triton X-100 in PBS were used to block the nonspecific antigen for 60 min at RT. Then, the slides were incubated with the primary antibodies (WTAP, 1:200, Santa Cruz; Amelx, 1:100, Amelx, Santa Cruz; SHH, Bio-Techne, 1:100) in blocking buffer overnight at 4 °C. On the following day, after three times (10 min/times) wash in PBS with 0.1% Triton X-100, Alexa Fluor 488-/594-conjugated donkey secondary antibody (Jackson Immuno Research Laboratories; 1:500) were then added on the slides. Incubated at RT for 60 min, the slides were washed in PBS, rinsed quickly in pure ethanol, and mounted in Prolong Gold Antifade medium with 4′,6-diamidino-2-phenylindole (Molecular Probes). Finally, the results were analyzed by fluorescence microscopy (Olympus).

### Masson staining

The prepared sections were deparaffinized, hydrated, and washed. The staining procedures were operated according to the manufacturer protocol (Co, Ltd Maixin, MST-8003). Compound dye solution supplied in the kit was used to nuclear staining for 5 min. Next, the slides were washed by clean water. Followed by phosphato-molybdic acid staining (5 min) and immersed in 2% aniline blue solution for 5 min. Then, the slides were differentiated with 1% differentiation solution for 40 s, washed in water, dehydrated by graded ethanol, and mounted with neutral gum. Enamel tissues in the microscopic performance of Masson staining usually were red, dentin and bone were blue, and muscles were purple. All of the pictures in our study were taken by Nikon camera.

### BrdU labeling

BrdU was injected into the pregnant mouse or newborn mouse (50 μg/g of body weight), 2 h prior to sacrificing the mouse. Samples were embedded as described previously. After hydration through graded ethanol, the sections were incubated in 0.1% PBST for 30 min and incubated in 2M HCl solution for 30 min under 37 °C, followed neutralization by boric acid for 10 min at RT. The sections were blocked with normal donkey serum at RT for 60 min before incubation in BrdU antibody (1:200, Sigma–Aldrich) overnight at 4 °C. Sections were washed in PBST three times. Second antibody donkey antimouse were diluted 1:500 for 60 min at RT.

### Cell culture

ALCs were gifts from Pro. Wantao Chen’s lab (Shanghai Key Laboratory of Stomatology). Extracting method was described in previous study (22). ALC cells were cultured in Dulbecco's modified Eagle's medium supplied with 10% fetal bovine serum in 37 °C, 5% CO^2^. Cells were plated in 6-well plate about 70% confluent. Change to fresh culture media containing 8 μg/ml polybrene. Incubate cells at 37 °C, 5% CO^2^ overnight. Change to fresh media 6 h after infection. Infected cells were selected by culture medium with 2 μg/ml puromycin 24 h after infection.

### Plko.1-shRNA construction

Plko.1-shRNA plasmid was constructed according to the manufacturer (https://www.addgene.org/tools/protocols/plko). Sequence of oligos was listed in Table S2.

### Real-time PCR and m6A-RIP-qPCR

Total RNA for dot blot was isolated from enamel organ and ALC cells and was extracted with Trizol reagent (Invitrogen), then mRNA was extracted using GenElute mRNA miniprep (Sigma–Aldrich). m6A mRNA immunoprecipitation (m6A-RIP) was performed using a GenSeq m6A RNA IP kit. The RNAs were reverse transcribed using a PrimeScript RT reagent kit (Takara) and further analyzed by quantitative PCR. Sequence of primers were listed in Table S3.

### Western blotting

Cells were harvested and lysed in SDS lysis; protein concentration from lysis supernatant was determined by the Bradford method. Forty micrograms protein of each sample were separated by SDS-PAGE under reducing conditions and transferred to polyvinylidene difluoride membrane for 45 min at 130 V. Membranes were saturated with 5% skimmed milk and incubated with antibodies (WTAP, 1:1000, Sant Cruz; GAPDH, 1:10,000, Santa Cruz) overnight at 4 °C. After washing with TBST (supplemented with 0.1% Tween-20), the membranes were incubated with peroxidase-conjugated IgG second antibody and rinsed with TBST, and finally developed using the ECL Western Blotting Analysis System (Shanghai Yeasen Co Ltd).

### Crystal violet staining and alizarin red staining

ALC cells were cultured in mineralization-inducing media containing 100 mmol/l ascorbic acid, 10 nmol/l dexamethasone, and 2 mmol/l b-glycerophosphate. After incubation for 3 weeks, cells were fixed with 4% paraformaldehyde and stained with 1% Alizarin Red solution for 30 min at RT.

### m6A dot blot assay

GenElute mRNA Miniprep Kit (Sigma) was used to extract mRNA from mandibular tissue and ALC cells according to manufacturer’s protocols. At embryonic stages E14.5, mice enamel organ was harvest. To ensure sufficient concentration of mRNA, the enamel organ of 20 biological replicates were pooled for each sample. Dots (50 ng mRNA per 1.5 μl dot) were applied to an Amersham Hybond-N+ membrane (GE Healthcare). After complete drying the mRNA sample, a UV Stratalinker 2400 was used to crosslink mRNA to the membrane by running the autocrosslink program at 3000 kJ. After three times washing in PBST (0.1% Tween-20 in PBS), the membrane was blocked in 5% skim milk in PBST for 2 h. Then, repeating three times PBST wash, the mRNA crosslinked membrane was incubated with primary anti-m6A antibody (212B11, Synaptic Systems) at 1:1000 dilution for 2 h at RT. Repeating three washes in PBST, horseradish peroxidase–conjugated anti-mouse IgG secondary antibody (Jackson ImmunoResearch) was used to incubate for 1 h at RT. Finally, after three washes, the membrane was visualized. Additionally, before incubation with antibodies, the membrane was stained with 0.02% methylene blue in 0.3 M sodium acetate (pH 5.2) to confirm equal mRNA loading. Next, quantified m6A levels were normalized to amount of mRNA loaded. For each time point, three biological samples in technical duplicates were used.

### Ultraperformance liquid chromatography-MS/MS analysis of m6A levels

Nuclease P1 (1 U; Sigma) in 20 μl of buffer, which contained 10 mM of NH_4_Ac (pH 5.3), was used to digest mRNA at 42 °C for 4 h. Then, 100 mM NH_4_HCO_3_ and alkaline phosphatase (0.5 U) were then added to about 50 to 100 ng purified mRNA for another incubation at 37 °C for 4 h. Next, the supernatant of digested sample was collected by centrifugation (4 °C, 13,000 rpm, 20 min) and then injected into ultraperformance liquid chromatography-MS/MS. Ultraperformance liquid chromatography (SHIMADZU) equipped with ZORBAX SB-Aq column (Agilent) was used to separate the nucleosides. Then, Triple Quad 5500 (AB SCIEX) in positive ion multiple reaction-monitoring mode was used to detect the nucleosides. According to nucleoside-to-base ion mass transitions, the modifications were quantified: *m/z* 268.0 to 136.0 for A and *m/z* 282.0 to 150.1 for m6A. Pure nucleosides were used to generate standard curves. Then, the concentrations of A and m6A in the sample were calculated. Finally, the percentage of total unmodified A represents the level of m6A.

### mRNA stability assay

About 5 × 10^5^ ALC cells with stably expressed sh*Wtap* or shNegative control were seeded into 6-well plates. After 24 h, cells were treated with 5 μg/ml actinomycin D and collected at indicated time points. The total RNA was extracted by TRIzol protocol (Takara) and analyzed by RT-PCR. Half-life of mRNA was analyzed by GraphPad 8.3.0 (GraphPad Software Inc) according to previously published paper.

## Data availability

All data are contained within the article.

## Supporting information

This article contains supporting information.

## Conflict of interest

The authors declare that they have no conflicts of interest with the contents of this article.

## Figures and Tables

**Figure:**
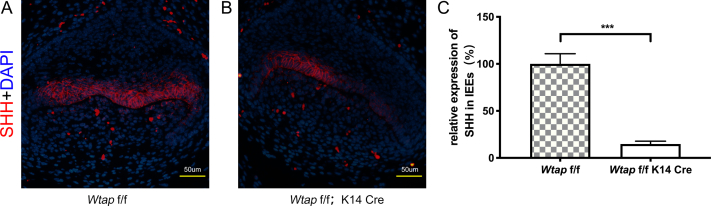
Supplemental Figure S5

## Supporting information

Supplemental Figure Captions and Tables S1–S3
