# Supplementary material for: N6-methyladenosine (m6A) RNA methylation mediated by methyltransferase complex subunit WTAP regulates amelogenesis
Source: J Biol Chem. 2022 Nov 17;298(12):102715. doi: 10.1016/j.jbc.2022.102715 (PMC9791132; doi:10.1016/j.jbc.2022.102715)
Supplement: Supplemental Figure Captions and Tables S1–S3 [file mmc1.docx]

**Supplymentary Figures legend**

Supplementary Figure 1. WTAP is widely expressed at early stage of tooth development. A: E11.5; B: E13.5; C: E14.5; D: E16.5; E: E18.5; F: P0. (dpn, days postnatal; dpc, days postcoitus); G: Statistics analysis result of relative expression of WTAP.

Supplementary figure 2. Expression of WTAP protein in human reduced dental epithelium of unerupted 3rd molar tooth. A, DAPI staining; B, K14 staining; C, WTAP staining; D, merge.

Supplementary figure 3. PCR results of conditional knock-out mice genotype identification

Supplementary figure 4. Knockdown of *Wtap* in ameloblast-lineage cell (ALC) resulted decreased cell proliferation and differentiation in vitro. A: Real-time PCR showed lower expression of *Wtap* in ameloblast cell; B: Western blot confirmed knockdown efficiency of *Wtap* in ameloblast cell; C: Crystal violet staining showed lower proliferation ability after *Wtap* knockdown; D: Alizarin red staining showed decreased cell differentiation ability of ameloblast cell.

Supplementary figure 5.

Decreased expression of SHH protein in dental inner enamel epithelial cells of cKO mandibular first molar at E16.5. A: SHH expression in dental IEEs from control mandibular first molar was detected by immunofluorescence staining; B: SHH expression in dental IEEs from mutant mandibular first molar was detected by immunofluorescence staining; C: statist analysis of SHH expression in dental IEEs of mandibular first molar. (dpc, days postcoitus)

Supplementary figure 6. Tooth development showed no malformation after WT1 knockout in mice dental epithelial tissue. A: WT1 expression was detected by immunofluorescence staining in dental tissue at E16.5; B; mouse testis was used as positive control to test WT1 antibody; C, D: photo of incisor from *Wt1* ^f/f^ *and Wt1* ^f/f^; K14 Cre mice respectively.

Supplementary Table 1. Primers for genotype PCR.

| Primer name | Sequence(5’-3’) |
| --- | --- |
| *Wtap*-Forward | CCT CAA ATT AGA GAT CTC CCT TAG TC |
| *Wtap*-Reverse | GCT TGG CAC CTT AGC AAG AC |
| *Cre*- Forward | CGATGCAACGAGTGATGAGG |
| *Cre*-Reverse | CGCATAACCAGTGAAACAGC |

Supplementary Table 2. Sequence of oligos for cloning into pLKO.1.

| Primer name | Sequence(5’-3’) |
| --- | --- |
| *Wtap*_sh1_Forward | CCGGCCGATTGAGTGAAACAGATTTGTTAATATTCATAGCAAGTCTGTTTCACTCAGTCGGTTTTTG |
| *Wtap*_sh1_Reverse | AATTCAAAAACCGATTGAGTGAAACAGATTTGCTATGAATATTAACAAGTCTGTTTCACTCAGTCGG |
| Wtap_sh2_Forward | CCGGGCAAGAGTGTACCACTTAAATGTTAATATTCATAGCATTTGAGTGGTGCACTCTTGCTTTTTG |
| *Wtap*_sh2_Reverse | AATTCAAAAAGCAAGAGTGTACCACTTAAATGCTATGAATATTAACATTTGAGTGGTGCACTCTTGC |

Supplementary Table 3. Primers for quantitative PCR.

| Primer name | Sequence(5’-3’) |
| --- | --- |
| *Wtap*-Forward | GCAAGATGACCAACGAAGAAC |
| *Wtap*-Reverse | CCAGTCACATCGTTTGAATTAAG |
| *Shh*-Forward | AAAGCTGACCCCTTTAGCCTA |
| *Shh*-Reverse | TTCGGAGTTTCTTGTGATCTTCC |
| *Gapdh*-Forward | ACCACAGTCCATGCCATCAC |
| *Gapdh*-Reverse | TCCACCACCCTGTTGCTGT |
